# Wearing Flannel

**Published:** 1859-01

**Authors:** 


### WEARING FLANNEL.

The very best thing that can be worn next the skin, in summer as well as winter, is common woolen flannel. One color has no advantage over another, except that white is more agreeable to the sight, it is more likely to "full up" in washing; but this may be almost entirely prevented, if done properly. Pour boiling hot strong soapsuds on the garment in a tub, let it alone until the hand can bear the water, then pour off and add clean water, boiling hot, let this stand also as before; pour off and add more boiling clean water, and when cool enough, merely squeeze the garment with the hands—no wringing or rubbing. Stretch it immediately on a line in the hot sun, or before a hot fire, and as the water settles at the most dependant part of the garment, press it out with the hand, and be careful to stretch the fabric as soon as the water is squeezed out, aiming as much as possible to keep the flannel hot until it is dry. If woolen garments are treated literally as above, they will remain pliable and soft until worn out.

Recent scientific experiments, carefully conducted, prove the truth of the popular sentiment, that woolen flannel is the best fabric to be worn next the skin, as it absorbs more moisture from the body than any other material, and by so doing, keeps the body more perfectly dry. Cotton absorbs the least, hence the perspiration remains more on the skin, and being damp, the heat of the body is rapidly carried off by evaporation and suddenly cools when exercise ceases, the ill effects of which no intelligent mind need to be reminded of. Hence it is, that the common observation of all nations leads them to give their sailors woolen flannel shirts for all seasons and for all latitudes, as the best equalizers of the heat of the body.

